# Removal of inorganic impurities from wastewater after production of soda ash on selected sorbents

**DOI:** 10.1038/s41598-020-61429-w

**Published:** 2020-03-12

**Authors:** Adam Gołub, Janina Piekutin

**Affiliations:** Bialystok University of Technology, Department of Technology in Engineering and Environmental Protection, Wiejska 45A, 15-351 Białystok, Poland

**Keywords:** Pollution remediation, Environmental impact, Hydrology

## Abstract

The soda ash industry is a part of the chemical industry, which is responsible for the production of sodium carbonate, calcium chloride, absorbent masses, evaporated wet salt, food salt, pickling salt or salt tablets. During manufacturing of those products, strongly alkaline wastewater is generated. It could be characterised by a high electrolytic conductivity and concentration of ions: chlorides, sulfates, phosphates, calcium, sodium, magnesium, potassium and ammonium. The aim of the research was to test the effectiveness of removing sodium, potassium, calcium, magnesium and ammonium from wastewater after production of soda ash by three sorbents: Halosorb, Compakt and Damsorb K. The process was carried out using dynamic method with different flow of wastewater through the column with sorbent. It allowed to reduce concentrations of all cations tested. Moreover, it was found that sorbent type did not significantly affect the removal of any of the ions, but the deposit load had significant impact on the removal of all ions tested.

## Introduction

Protection of water against pollution should be associated with rational management of water resources, restoration of environment to the required state and prevention from pollution. The strict control of pollution at the source is becoming an increasingly important form of protection^1^. In the industry it is closely associated with the issue of cleaner production, which requires integrated actions in relation to processes and products aimed, on the one hand, at increasing the production efficiency, and, on the other hand, to reducing the risk for people and aquatic environment. This aims, among others, at preventing and reducing the source of sewage and solid waste, as well as to save water, energy and other natural resources during production processes. Elimination of toxic and raw materials from production processes plays a crucial role as an important stage of preventing them from entering the water resources^1–6^.

Plants involved in the production of soda ash belong to the chemical industry. Most often they use the Solvay method as their production process, which is associated with the formation of saline waste^7–10^. Limiting the negative impact on environment consists in pre-treating the post-production sewage and restricting its contact with underground water^11^. Striving to improve water quality, reduce costs associated with environmental protection and eliminate pollution at the source according to cleaner production assumptions causes that industrial plants are actively seeking innovative and effective ways to protect water resources. Individual ions present in such wastewater could be removed from wastewater among others in the processes of nanofiltration, reverse osmosis, precipitation, biological treatment, ion exchange resins^12–19^. However, most of them are expensive and ineffective in relation to all mentioned ions simultaneously. One of the promising methods in the treatment of post-production sewage has become the use of easily accessible, simple to use, low-cost and, importantly, environmentally non-toxic sorbents^7,20^.

The aim of the research was to evaluate the effectiveness of removing selected cations from wastewater after production of soda ash in the sorption process.

## Methods

Tests on cation removal from sewage after soda ash production were carried out using 3 sorbents. As a sorbent, processed halloysite Halosorb and calcined diatomaceous earth Compakt and Damsorb K were used. The above materials were chosen because they are a compromise between the requirements of industrial plants (they are cheap, easily accessible, easy to use) and environmental protection requirements (easy sorbent utilization, no threat to the environment due to their composition). Physicochemical properties of the materials used are shown in Table 1.

**Table 1:** Physicochemical properties of sorbents used for testing.

Parameter	Halosorb	Compakt	Damsorb K
Grain diameter [mm]	0.2–3	0.3–0.7	0.3–1.5
Average loose density [g∙dm^−3^]	680	525	429
Chemical composition	- SiO_2_ (40%) - Al_2_O_3_ (33%) - Fe_2_O_3_/FeO (8%) - TiO_2_ (2%) - CaO (1.3%) - MgO (0.5%) - Na_2_O (0.1%) - K_2_O (0.1%)	- SiO_2_ (75%) - Al_2_O_3_ (10%) - Fe_2_O_3_ (7%) - MgO (2%) - TiO_2_ (1%) - CaO (1%) - K_2_O + Na_2_O (2%)	- SiO_2_ (71%) - Al_2_O_3_ (10.5%) - Fe_2_O_3_ (8.4%) - CaO (2.5%) - K_2_O + Na_2_O (2.1%) - MgO (1.6%) - TiO_2_ (1.4%)
pH (10% water suspension)	7.0	5.5	5.5
Absorptivity [%]	80–120	90–110	90–130

Industrial wastewater generated in the production of soda ash is a liquid waste characterized by high pH and concentration of tested cations. Wastewater was collected as a waste mixed from two production plants: Soda Mątwy in Inowrocław and Janikosoda in Janikowo. The tests included concentrations of five cations: ammonium, sodium, calcium, potassium, magnesium. The above plants did not agree to publish the quality of wastewater used for the research.

Cation removal process was carried out using the dynamic method. The subsequent glass columns with a diameter 60 mm and length 320 mm were filled with a portion of 250 g of each of three sorbents (first column with Halosorb, second one with Compakt and third one with Damsorb K). In order to remove the physical impurities present on the sorbent, they were rinsed with distilled water (in the amount of double volume of the column) prior to the main process. The research was carried out in four series using different loads of each of them (Table 2). Everytime first 250 cm^3^ of wastewater flowed throught the column and then 250 cm^3^ was collected as a sample. Confirmation of results repeatability required conducting the experiment twice. After experiment, the sorbent columns were again rinsed with distilled water.

**Table 2:** Deposit loads in subsequent series of tests.

Series of tests	Deposit load [m^3^ ∙ m^−2^ ∙ h^−1^]
I.	1.937
II.	1.628
III.	0.969
IV.	0.579

Determinations of the analyzed cations concentrations were performed on a Thermo Scientific ICS 5000+ ion chromatograph. The results were statistically evaluated using the following tests: Shapiro-Wilk, Scheffe and Kruskal-Wallis.

## Results

### Sodium

**Figure 1:**
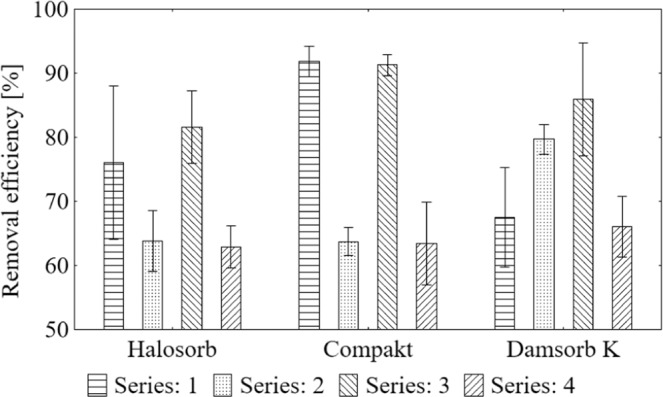
Average value and standard deviation of sodium removal degree during the sorption process on the tested materials: Halosorb, Compakt and Damsorb K.

Degree of sodium removal using sorbents (Fig. 1) is presented as the mean result with the standard deviation. Halosorb reduced the ion concentration from 62.94% to 81.59%, Compakt - from 62.94% to 91.86%, while Damsorb K - from 66.05% to 85.88%.

#### Checking the normality of variables distribution

The Shapiro-Wilk test was used to check the normality of variables distribution. At the significance level of *α* = 0.05, the probability level was determined as *p* = 0.00211. Due to the fact that the probability level *p* had a value lower than the significance level *α*, the null hypothesis about the normality of the distribution should have been rejected.

#### Assessment of sorbent type influence on the degree of sodium removal

The Kruskal-Wallis test made it possible to conclude that with the probability level of *p* = 0.4025, there was no reason to reject the null hypothesis that the factor did not influence the results of the experiment. The type of sorbent did not therefore differentiate the degree of sodium removal from wastewater.

#### Assessment of the impact of deposit load on the degree of sodium removal

Results from the Kruskal-Wallis test indicated that with the probability level of *p* = 0.0017, the null hypothesis about the lack of influence of the factor on the test results should have been rejected. Based on the analysis of variance, it was determined that only the load on the deposit had significant impact on the test results.

In order to select the most effective deposit loading for each sorbent and divide sorbents’ efficacy into homogeneous groups to find out if the differences are statistically significant, a Scheffe *post-hoc* test was carried out (Table 3). On this basis, the highest efficiency of Halosorb in the III series of tests (82%) was determined, which significantly differed from other series, for which it was 76% in I, and 64% and 63%, respectively in II and IV series. In the case of Compakt, the best removal effect was observed in the I and III series of tests (91–92%), while in the II and IV series, it was definitely smaller (63–64%). Damsorb K removed sodium in the highest degree in the III series (86%), slightly lower in the II series (80%), whereas in the remaining series, the level of reduction was even lower (66–68%).

**Table 3:** Scheffe test results of the average degree of sodium removal in subsequent series of measurements grouped by the type of sorbent.

Group	Series (removal efficiency [%])		
	Halosorb	Compakt	Damsorb K
1	III (81.59)	III (91.29), I (91.86)	III (85.88)
2	I (76.05)	IV (62.94), II (63.84)	II (79.70)
3	IV (62.94), II (63.84)		IV (66.05), I (67.53)

### Calcium

**Figure 2:**
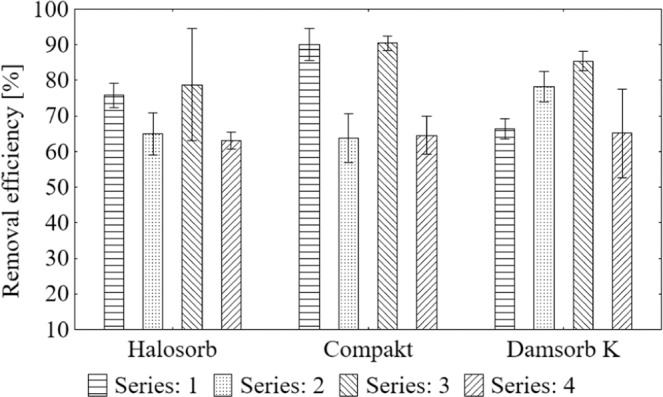
Average value and standard deviation of calcium removal degree during the sorption process on the tested materials: Halosorb, Compakt and Damsorb K.

Degree of calcium removal in the sorption process (Fig. 2) is presented as the mean result with standard deviation. The ion removal by Halosorb ranged from 63.05% to 78.75%, Compakt from 63.79% to 90.42%, and Damsorb K from 65.16% to 85.45%.

#### Checking the normality of variables distribution

To verify the normality of the distribution of variables, the Shapiro-Wilk test was applied again. Assuming the significance level *α* = 0.05, the probability level was determined as *p* = 0.00145. Due to lower value of the probability level *p* than significance level *α*, the null hypothesis on normality of the distribution had to be rejected.

#### Assessment of sorbent type influence on the degree of calcium removal

The Kruskal-Wallis test allowed to conclude that with the probability level *p* = 0.4371, there was no reason to reject the null hypothesis that the factor did not influence the experimental results. The type of sorbent did not therefore differentiate the degree of calcium removal from wastewater.

#### Assessment of the impact of deposit load on the degree of calcium removal

Results from the Kruskal-Wallis test indicated that with the probability level *p* = 0.0017, the null hypothesis about the lack of influence of the factor on the test results should have been rejected. Based on the analysis of variance, it was determined that only the load on the deposit had a significant impact on the test results.

The most effective deposit loads for each sorbent was selected after the Scheffe *post-hoc* test (Table 4). The highest efficiency of Halosorb was observed in III and I series of tests (79% and 76%), while in II and IV, it was 63–65%. In the case of Compakt, the best removal effect was also recorded in III and I series of tests (in both cases about 90%), while in the IV and II series, it was definitely smaller (64–65%). Damsorb K removed calcium in the highest degree in the III series (85%), whereas in the II series, the degree was slightly lower (78%), and in the I and the IV - definitely the lowest (65–66%).

**Table 4:** Scheffe test results of the average degree of calcium removal in subsequent series of measurements grouped by the type of sorbent.

Group	Series (removal efficiency [%])		
	Halosorb	Compakt	Damsorb K
1	I (75.74), III (78.75)	I (89.98), III (90.42)	III (85.45)
2	IV (63.05), II (64.85)	II (63.79), IV (64.58)	II (78.31)
3			IV (65.16), I (66.46)

### Potassium

**Figure 3:**
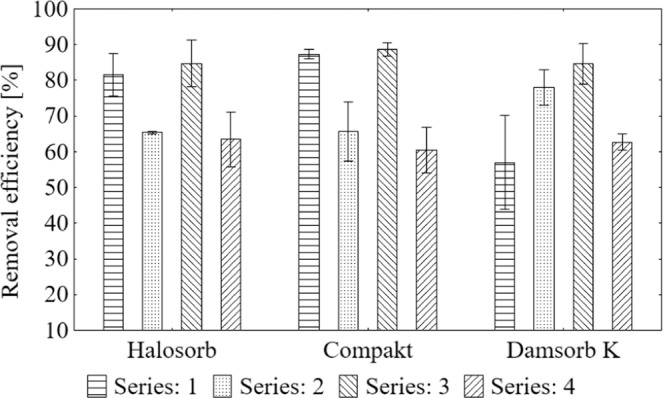
Average value and standard deviation of potassium removal degree during the sorption process on the tested materials: Halosorb, Compakt and Damsorb K.

Degree of potassium removal on the tested sorbents (Fig. 3) is presented in the form of average result with standard deviation. Potassium was removed on Halosorb in the range from 63.43% to 84.71%, on Compakt - from 60.52% to 88.55%, while on Damsorb K - from 57.00% to 84.53%.

#### Checking the normality of variables distribution

Checking the normality of the variable distribution required the use of Shapiro-Wilk test. Level of probability defined at the significance level *α* = 0.05 was *p* = 0.00389, which suggested rejecting the hypothesis of distribution normality (*p* < *α*).

#### Assessment of sorbent type influence on the degree of potassium removal

The Kruskal-Wallis test made it possible to conclude that with the probability level *p* = 0.357, there was no reason to reject the null hypothesis that the factor did not affect the results of experiment. Type of sorbent did not therefore differentiate the degree of potassium removal from wastewater.

#### Assessment of the impact of deposit load on the degree of potassium removal

Results from the Kruskal-Wallis test indicated that with the probability level of *p* = 0.0045, the null hypothesis about the lack of influence of the factor on the test results should have been rejected. Based on the analysis of variance, it was determined that only the load on the deposit had significant impact on the test results.

The Scheffe test was used to select the most effective deposit load (Table 5). Halosorb achieved the best result in the III series of tests (85%), slightly worse in the I series (82%), and by far the worst in the II and IV series (63–65%). The effectiveness of Compakt was the highest in the III and I series of tests (89% and 87%, respectively); it decreased in the II series (66%), while in the IV series, it was the lowest (61%). The most diversified results were obtained for Damsorb K, which in the III series demonstrated the effectiveness of reducing potassium at 85%, which decreased as follows: the II series (78%), the IV series (63%) and the I series (57%).

**Table 5:** Scheffe test results of the average degree of potassium removal in subsequent series of measurements grouped by the type of sorbent.

Group	Series (removal efficiency [%])		
	Halosorb	Compakt	Damsorb K
1	III (84.71)	I (87.34), III (88.55)	III (84.53)
2	I (81.56)	II (65.70)	II (77.99)
3	IV (63.43), II (65.33)	IV (60.52)	IV (62.65)
4			I (57.00)

### Magnesium

**Figure 4:**
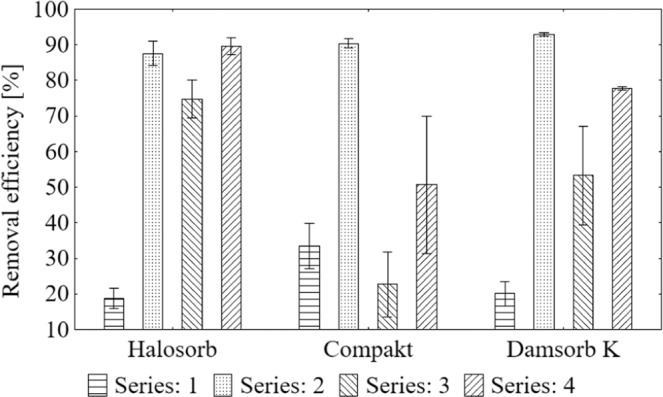
Average value and standard deviation of magnesium removal degree during the sorption process on the tested materials: Halosorb, Compakt and Damsorb K.

Degree of magnesium removal during sorption on the tested materials (Fig. 4) is presented as the mean result with standard deviation. The degree of removal by Halosorb was at the level from 18.84% to 89.58%, by Compakt - from 22.75% to 90.37%, while by Damsorb K - from 20.16% to 92.90%.

#### Checking the normality of variables distribution

Normality of the variable distribution was examined using the Shapiro-Wilk test. Assuming the significance level *α* = 0.05, the probability level was determined as *p* = 0.00209. Due to lower value of the probability level *p* than the significance level *α*, the hypothesis about the normality of distribution had to be rejected.

#### Assessment of sorbent type influence on the degree of magnesium removal

The Kruskal-Wallis test allowed to conclude that with the probability level *p* = 0.7925, there was no reason to reject the null hypothesis that the factor did not influence the experimental results. Type of sorbent did not therefore differentiate the degree of magnesium removal from wastewater.

#### Assessment of the impact of deposit load on the degree of magnesium removal

Results of the Kruskal-Wallis test indicated that with the probability level of *p* = 0.0004, the null hypothesis about the lack of influence of the factor on the test results should have been rejected. Based on the analysis of variance, it was determined that only the load on the deposit had significant impact on the test results.

The Scheffe *post-hoc* test was used for each sorbent to select the most effective deposit load (Table 6). On this basis, the highest efficiency of Halosorb in the IV series of tests (90%) was determined, which significantly differed from the other series, where in the II it was 88%, in III - 75%, while in I only 19%. In the case of Compakt, the best removal effect was observed in the II series of tests (90%) and lower in IV (51%), I (33%) and III series (23%). Damsorb K removed magnesium in the highest degree in the II series (93%); in the IV series the level of reduction was 78%, in the III series - 53% and in the I series - 20%.

**Table 6:** Scheffe test results of the average degree of magnesium removal in subsequent series of measurements grouped by the type of sorbent.

Group	Series (removal efficiency [%])		
	Halosorb	Compakt	Damsorb K
1	IV (89.58)	II (90.37)	II (92.90)
2	II (87.55)	IV (50.67)	IV (77.74)
3	III (74.77)	I (33.43)	III (53.26)
4	I (18.84)	III (22.75)	I (20.16)

### Ammonia ion

**Figure 5:**
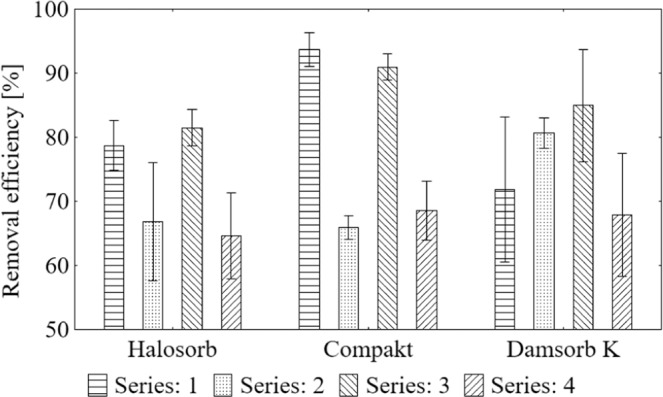
Average value and standard deviation of ammonia ion removal degree during the sorption process on the tested materials: Halosorb, Compakt and Damsorb K.

The degree of removal of the ammonium ion (Fig. 5) is shown as the average result, also indicating the standard deviation. Halosorb removal effect ranged from 64.61% to 81.51%, Compakt from 65.88% to 93.67%, and Damsorb K from 67.89% to 84.98%.

#### Checking the normality of variables distribution

The Shapiro-Wilk test was used to check the normality of variables distribution. At the significance level of *α* = 0.05, the probability level was determined as *p* = 0.01773. The probability level *p* had lower value than the significance level *α*, thus the null hypothesis about the normality of the distribution should have been rejected.

#### Assessment of sorbent type influence on the degree of ammonia removal

The Kruskal-Wallis test allowed to conclude that with the probability level *p* = 0.3597, there was no reason to reject the null hypothesis that the factor did not affect the results of experiment. Type of sorbent did not therefore differentiate the degree of magnesium removal from wastewater.

#### Assessment of the impact of deposit load on the degree of ammonia removal

Results of the Kruskal-Wallis test indicated that with the probability level of *p* = 0.0021, the null hypothesis about the lack of influence of the factor on the test results should have been rejected. Only the load on the deposit had significant impact on the degree of ammonium concentration reduction.

In order to select the most effective deposit load for each sorbent, a Scheffe *post-hoc* test was carried out (Table 7). Based on it, the highest efficiency of Halosorb in the III and I series of tests was determined (82% and 79%, respectively), which significantly differed from the other series, where in II series, it amounted to 67%, and in IV - 65%. In the case of Compakt, the best removal effect was observed in the I series of tests (94%), while in others, it was already lower: in the III series - 91%, in the IV - 69% and in the II - 66%. Damsorb K removed the ammonium ion to the highest degree in the III series (85%), in the II series in 81%, while in other series, the level of reduction was lower (in I - 72% and in IV - 68%).

**Table 7:** Scheffe test results of the average degree of ammonia ion removal in subsequent series of measurements grouped by the type of sorbent.

Group	Series (removal efficiency [%])		
	Halosorb	Compakt	Damsorb K
1	I (78,71), III (81,51)	I (93,67)	III (84,98)
2	IV (64,61), II (66,81)	III (90,98)	II (80,65)
3		IV (68,53)	IV (67,89), I (71,85)
4		II (65,88)	

## Summary and Discussion

The paper presents results of research on the removal of ammonium, sodium, calcium, potassium and magnesium ions from wastewater after soda ash production, by sorption on three sorbents: Halosorb, Compakt and Damsorb K. Four series of tests were carried out using the dynamic method, each applying different load on the deposit: 1.937 m^3^ ∙ m^−2^ ∙ h^−1^, 1.628 m^3^ ∙ m^−2^ ∙ h^−1^, 0.969 m^3^ ∙ m^−2^ ∙ h^−1^, 0.579 m^3^ ∙ m^−2^ ∙ h^−1^. Confirmation of results repeatability required conducting the experiment twice. The exact interpretation of the obtained results was made due to the use of statistical tests: Shapiro-Wilk, Scheffe and Kruskal-Wallis.

After performing detailed analyses, it was found that the results obtained for individual sorbents are not significantly different, which indicates that the type of deposit used does not affect the degree of removal of any of the examined ions. A similar effect can be obtained using any of them.

In the case of all cations, it was found that the load on the deposit significantly influences the experimental results. Sodium removal at Halosorb and Damsorb K was most effective at the load 0.969 m^3^ ∙ m^−2^ ∙ h^−1^ (82% and 85% respectively), while on Compakt at the load 1.937 m^3^ ∙ m^−2^ ∙ h^−1^ (94%). At the same time, it was observed that for all materials, the lowest effect was achieved with the deposit load of 0.579 m^3^ ∙ m^−2^ ∙ h^−1^. For comparison, scientific reports state about reducing the sodium concentration by about 10–60% using nanofiltration^12,15^. Similarly, the concentration of this ion was lowered using carbon nanotube sheets as a sorbent even by several dozen percent^21^ and up to 70% using functionalized graphene sheets^22^. Application of reverse osmosis could reduce content of sodium even by more than 98.5%^14,17,19^. The use of a precipitation process, e.g. with amine solvents such as isopropylamine, was not effective - the result was close to 0%^13^. Comparing to this, effect achieved in decreasing the sodium concentration by not modified sorbents in this research seems promising, especially considering its very high concentration in wastewater.

On the basis of subsequent tests, it was recorded that calcium was removed most effectively by all materials at the deposit load of 0.969 m^3^ ∙ m^−2^ ∙ h^−1^ (79–90%). The conducted research indicated a reduction in calcium concentration by about 40–80% applying nanofiltration^12,19^, even 90% on nanofibrous mats^23^ or over 99% when using reverse osmosis^17,19,24^. The use of precipitation with amine solvents, such as isopropylamine, allowed almost complete removal of calcium from the sample^13^. Results obtained during presented tests are similar to most effective methods described in scientific literature, therefore further research would be justified.

All sorbents showed the highest efficiency of lowering potassium concentration at the deposit load 0.969 m^3^ ∙ m^−2^ ∙ h^−1^ and it varied from 85% to 89%. Using nanofiltration, the effect of about 90% removal was achieved^15^. The use of reverse osmosis to remove potassium allowed to reduce its concentration by up to 97%^17,25^. Biological treatment on constructed wetlands decreased potassium concentration for almost 60%^18^. In this case results obtained in the experiment are similar to effects of nanofiltration, what suggests potential of sorbents in further study.

The ammonium ion on Halosorb and Damsorb K was most effectively removed at load 0.969 m^3^ ∙ m^−2^ ∙ h^−1^ (82% and 85% respectively), while on Compakt at the load of 1.937 m^3^ ∙ m^−2^ ∙ h^−1^ (93%). The membrane contactors and sorbent clinoptilolite are very effective in removing the ammonium ion from water. After their application, the removal rate reaches 100%^26–28^. Using sodium hydroxide modified zeolite mordenite allowed to achieve effect of about 80% ion removal^29^. Very similar effect – about 80% of removal – was obtained after application of ion exchange resins^16^. Ammonia removal degree on sorbents comparing to literature data is high enough to continue research on their application in salty industry wastewater treatment.

Magnesium removal was different than that of other ions. Halosorb showed the highest efficiency when load of the deposit was 0.579 m^3^ ∙ m^−2^ ∙ h^−1^ (90%), whereas Compakt and Damsorb K at the load of 1.628 m^3^ ∙ m^−2^ ∙ h^−1^ (91% and 93% respectively). Results published in the scientific literature refer to the reduction of magnesium concentration by 60–80% when using nanofiltration^12,19^ and by over 98% with the use of reverse osmosis^17,19,25^. Precipitation with amine solvents, e.g. isopropylamine, reduced magnesium concentration by about 30%^13^ and removal on activated coconut coir by about 50%^30^. As it is shown, degree of magnesium removal on sorbents is high comparing to another methods from literature and the tests should be continued.

It is worth noting that in most cases sorbents are the most effective at the load 0.969 m^3^ ∙ m^−2^ ∙ h^−1^. This may indicate that at higher flows the contact time of sorbent and wastewater is too short to remove ions to a high degree, at smaller flows however, due to the large amount of suspension in the wastewater, sorbent pores are blocked and sorption capacity decreases. High removal degree at the load 1.937 m^3^ ∙ m^−2^ ∙ h^−1^ may be due to the fact that this was the first contact of sorbent and wastewater at first series of tests. The remaining series were carried out on sorbents used for testing and rinsed with distilled water, hence the removal effect is more similar to the real one that would appear in an industrial scale installation, where the sorbent would be used repeatedly^20^.

The obtained test results do not exclude the possibility of work on increasing the effectiveness of sorbents used in the treatment of wastewater from soda ash production. A number of factors important in the sorption process should be taken into account, such as: sorption capacity, pre-treatment of the material, process economics, material availability on the market, sorption mechanism, sorbent utilization or management after its saturation^20^. The sorption capacity can be increased by modifying the surface of sorbents^31^.

## Conclusions

The following conclusions were drawn from the conducted research:

- The type of sorbent used does not affect the degree of removal of the ions studied.
- Removal of sodium and ammonium ion is most effective at the loads of deposit 0.969 m^3^ ∙ m^−2^ ∙ h^−1^ and 1.937 m^3^ ∙ m^−2^ ∙ h^−1^.
- The degree of calcium and potassium removal is highest when using the deposit load of 0.969 m^3^ ∙ m^−2^ ∙ h^−1^.
- Concentration of magnesium is most effectively reduced at the deposit loads 1.628 m^3^ ∙ m^−2^ ∙ h^−1^ and 0.579 m^3^ ∙ m^−2^ ∙ h^−1^.

## References

[1] Gromiec, M., Sadurski, A., Zalewski, M., Rowinski, P. Hazards related to water quality. *Nauka* **1/2014**, 99–122 [in Polish] (2014).

[2] Harat, A., Grmela, A. The impact of mining waters of the Upper Silesian Coal Basin on changes in water quality in the Olza River in 2000-2007. *Monitoring Środowiska Przyrodniczego* **9**, 57–62 [in Polish] (2008).

[3] Mosiej, J., Komorowski, H., Kaczmarczyk, A., Suska, A. Impact of pollution discharged from the Łódź agglomeration on water quality in the rivers Ner and Warta. *Acta Sci Pol* **6(2)**, 19–30 [in Polish] (2007).

[4] Policht-Latawiec, A., Kanownik, W., Łukasik, D. Impact of point pollution on water quality in the San River. *Infrastruktura i ekologia terenów wiejskich* **1/IV/2013**, 253–269 [in Polish] (2013).

[5] Sukiennik, K. Problems of environmental management in enterprises. *Zeszyty Naukowe Politechniki Częstochowskiej* **8**, 38–49 [in Polish] (2012).

[6] Urbańska J, Urbański K (2012). Selected Aspects of Reclamation of Soda Waste Landfill Sites. Geomat. Environ. Eng..

[7] Gołub A, Piekutin J (2019). The Use of Sorbents in Removal of Selected Cations from Wastewater After Soda Ash Production. Proc..

[8] Matthews DA, Effler SW (2003). Decreases in pollutant from residual soda ash production waste. Water Air Soil. Pollut..

[9] Şener S (2008). Use of solid wastes of the soda ash plant as an adsorbent for the removal of anionic dyes: Equilibrium and kinetic studies. Chem. Eng. J..

[10] Steinhauser G (2008). Cleaner production in the Solvay Process: general strategies and recent developments. J. Clean. Prod..

[11] Siuta J (2014). Effectiveness of reclamation of soda waste disposal site at Janikowo using sewage sludge. Ecol. Eng..

[12] Bader MSH (2008). Analysis of the Paradox Valley brine desulfation by nanofiltration. Desalination.

[13] Bader MSH (2008). Innovative processes to desulfate the Paradox Valley brine. Desalination.

[14] Bobik, M., Labus, K. Mine water desalination in the industrial practice – state of the art and new challenges. *Przegląd górniczy* **4**, 99–105 (2014) [in Polish].

[15] Fatehizadeh A, Taheri E, Mehdi Amin M, Mohdavi M, Moradi N (2018). Sodium and potassium removal from brackish water by nanofiltration membrane: single and binary salt mixtures. Desalin water treat..

[16] Imchuen N, Lubphoo Y, Chyan JM, Padungthon S, Liao CH (2016). Using cation exchange resin for ammonium removal as part of sequential process for nitrate reduction by nanoiron. Sustain. Env. Res..

[17] Ogier, J., Doelchow, U., Warachim, Ł., Czarnocki, K. Reverse osmosis experiments as the fourth stage of filtration. *Technologia Wody* **2(64)**, 8–11 [in Polish] (2019).

[18] Sudarsan JS, Roy RL, Baskar G, Deeptha VT, Nithiyanantham S (2015). Domestic wastewater treatment performance using constructed wetland. Sustain. Water Resour. Manag..

[19] Turek, M., Laskowska, E., Mitko, K., Jakóbik-Kolon, A. Low energy utilization of saline mine waters in the integrated membrane-evaporating system. *Maszyny Górnicze* **36(1)**, 39–48 [in Polish] (2018).

[20] Kamiński, W., Tomczak, E. Low-cost sorbents application for water treatment. *Proceedings of ECOpole* **8(1)**, 189–194 [in Polish] (2014).

[21] Tofighy M, Mohammadi T (2010). Salty water desalination using carbon nanotube sheets. Desalination.

[22] Mishra AK, Ramaprabhu S (2011). Functionalized graphene sheets for arsenic removal and desalination of sea water. Desalination.

[23] Xiao S, Luo X, Peng Q, Deb H (2016). Effective removal of calcium ions from simulated hard water using electrospun polyelectrolyte nanofibrous mats. Fiber Polym..

[24] Subramania A, Jacangeloab JG (2014). Treatment technologies for reverse osmosis concentrate volume minimization: A review. Sep. Purif. Technol..

[25] Richards L, Richards BS, Schäfer AI (2011). Renewable energy powered membrane technology: Salt and inorganic contaminant removal by renewable energy powered nanofiltration/reverse osmosis. J. Memb. Sci..

[26] Mandowara A, Bhattacharya PK (2011). Simulation studies of ammonia removal from water in a membrane contactor under liquid–liquid extraction mode. J. Env. Manage.

[27] Margeta Karmen, Zabukovec Natasa, Siljeg Mario, Farkas Anamarija (2013). Natural Zeolites in Water Treatment – How Effective is Their Use. Water Treatment.

[28] Rezakazemi M, Shirazian S, Ashrafizadeh SN (2012). Simulation of ammonia removal from industrial wastewater streams by means of a hollow-fiber membrane contactor. Desalination.

[29] Soetardji JP (2015). Ammonia removal from water using sodium hydroxide modified zeolite mordenite. RSC Adv..

[30] Hettiarachchi E, Kottegoda N, Chandani Perera ADL (2016). Activated coconut coir for removal of sodium and magnesium ions from saline water. Desalin water treat..

[31] Loganathan P, Vigneswaran S, Kandasamy J, Bolan NS (2013). Removal and recovery of phosphate from water using sorption. Crit. Rev. Env. Sci. Tec..

